# Hemoglobin and adult height loss among Japanese workers: A retrospective study

**DOI:** 10.1371/journal.pone.0256281

**Published:** 2021-08-17

**Authors:** Yuji Shimizu, Hidenobu Hayakawa, Midori Takada, Takeo Okada, Masahiko Kiyama

**Affiliations:** Department of Cardiovascular Disease Prevention, Osaka Center for Cancer and Cardiovascular Diseases Prevention, Osaka, Japan; Nazarbayev University School of Medicine, KAZAKHSTAN

## Abstract

Height loss starting in middle age is reported to be associated with increased all-cause and cardiovascular mortality later in life. However, the mechanisms underlying this association are unclear. Hypoxia and oxidative stress, which are known causes of cardiovascular disease, could be reduced by hemoglobin. Therefore, hemoglobin could be inversely associated with height loss. However, high body mass index (BMI) is a known risk factor for intervertebral disc disorder, a known cause of height loss in adults. High BMI might confound the association between hemoglobin and height loss. Therefore, we performed analyses stratified by BMI status. To clarify the association between hemoglobin and height loss, we conducted a retrospective study of Japanese workers (6,471 men and 3,180 women) aged 40–74 years. Height loss was defined as being in the highest quintile of height decrease per year. In men overall and men with BMI <25 kg/m^2^, hemoglobin was significantly inversely associated with height loss; but no association was observed for men with high BMI (BMI ≥25 kg/m^2^) and for women. For men, after adjusting for known cardiovascular risk factors, adjusted odds ratios (ORs) and 95% confidence intervals (CIs) for height loss with each 1 standard deviation (SD) increase in hemoglobin (1.0 g/dL for men and 0.8g/dL for women) were 0.89 (0.83, 0.95) for men overall, 0.82 (0.75, 0.89) for men who do not have high BMI, and 1.01 (0.92, 1.12) for men with high BMI. For women, the corresponding values were 0.97 (0.89, 1.06), 0.98 (0.89, 1.09), and 0.93 (0.75, 1.15) respectively. Hemoglobin is significantly inversely associated with height loss in men who do not have high BMI, but not in men with high BMI or women. These results help clarify the mechanisms underlying height loss, which has been reported to be associated with a higher risk of mortality in adults.

## Introduction

Height loss starting in middle age is reported to be an independent risk factor for all-cause and cardiovascular mortality [1]. However, the mechanisms underlying the association between height loss and increased mortality are unclear. Understanding the mechanisms that cause height loss in middle age could help reduce mortality later in life.

Aging is a process that increases hypoxia and oxidative stress [2,3]. Hypoxia and oxidative stress, which are well known cardiovascular risk factors [4,5], have been reported to be associated with intervertebral disc disorder [6,7] and osteoporosis [8–10]. Since intervertebral disc disorder and vertebral fractures related to osteoporosis are well known causes of height loss in adults, hypoxia and oxidative stress might be associated with height loss.

Hypoxia causes oxidative stress [11]. The body produces erythropoietin, which activates hematopoiesis as an adaptation to hypoxia [12]. Erythropoietin increases hemoglobin concentrations [13]. Therefore, hemoglobin could act as an indicator of reduced hypoxia and oxidative stress. However, hematopoietic bone marrow activity declines with age [14,15]. Therefore, even though hemoglobin plays an important role in reducing hypoxia and oxidative stress, the process of aging lowers hemoglobin levels. In other words, hemoglobin could act as an indicator of variations in the aging process, such as height loss.

Previously, studies on the association between obesity (high body mass index [BMI]) and fracture have reported conflicting results [16]. However, another study reported participants with disc degeneration have higher BMI than participants without disc degeneration [17]. Another study reported that obesity is positively associated with intervertebral disc disorder [18]. Since obesity is reported to be positively associated with hemoglobin levels [19], high BMI could confound the association between hemoglobin and height loss by masking the beneficial effect of hemoglobin.

Therefore, we hypothesized that hemoglobin is inversely associated with height loss in participants with BMI <25 kg/m^2^. To clarify this association, we conducted a retrospective study of Japanese workers (6,471 men and 3,180 women) aged 40–74 years who participated in annual health check-ups between 2008 and 2018.

## Materials and methods

### Study population

The Ministry of Health, Labor and Welfare of Japan started specific medical examinations for cardiovascular disease prevention in 2008. In addition to physical examination and general laboratory tests of blood and urine samples, the medical examination contained a questionnaire about lifestyle and medical history.

**Fig 1:**
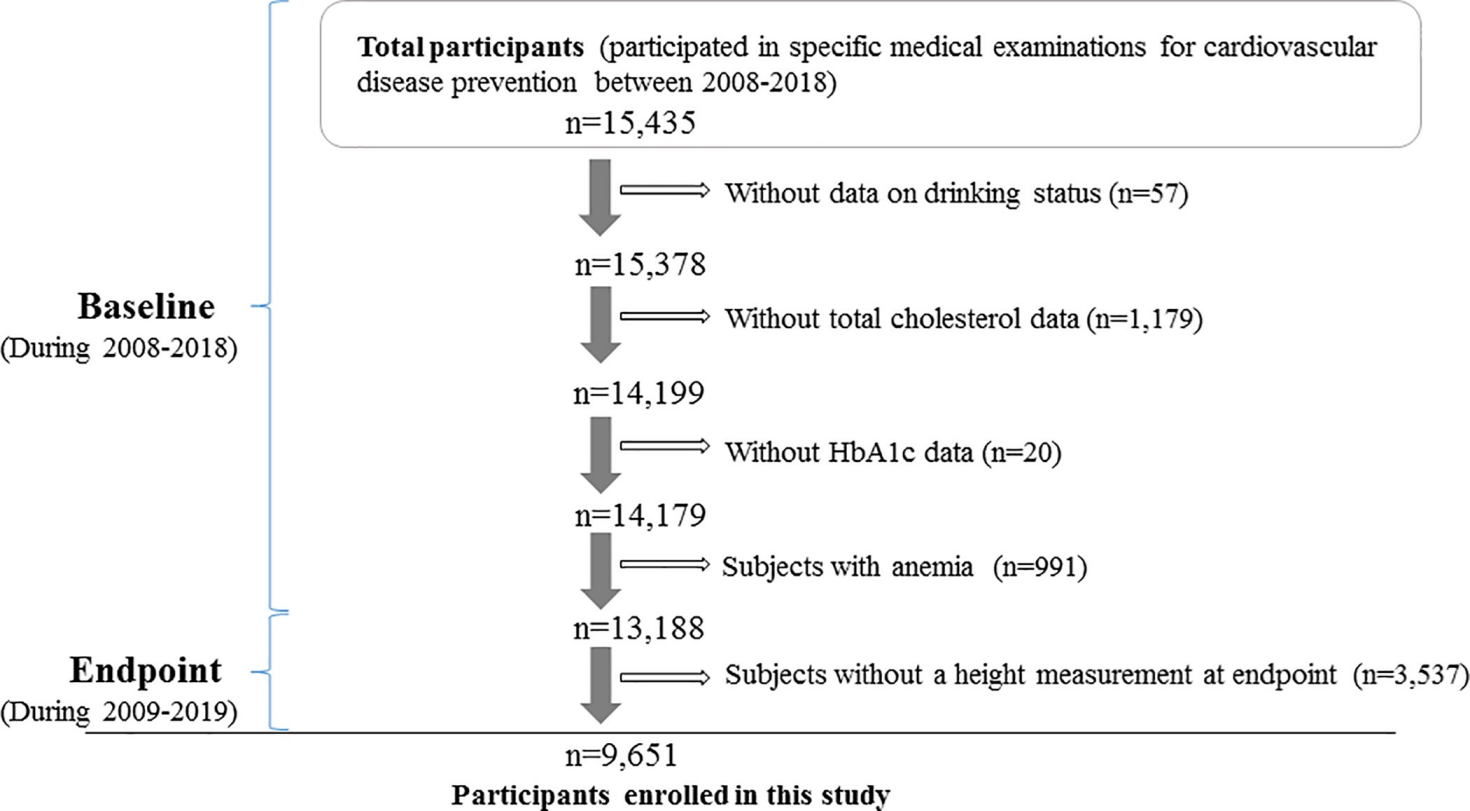
Demographics of study population.

Fig 1 shows the demographics of the study population. The present study population comprised 15,435 workers aged 40–74 years who participated in these specific medical examinations between 2008 and 2018 (baseline) at the Osaka Center for Cancer and Cardiovascular Diseases Prevention. Since the participants of this study were current workers who had the capacity to work, they might be relatively healthier than the general population. Furthermore, compared to the general population, the proportion of men might be higher because more men than women tend to become workers in Japanese society.

This study was approved by the ethics committee of the Osaka Center for Cancer and Cardiovascular Diseases Prevention (Project registration code: R2-Rinri-7). All procedures involving human participants were performed in accordance with the ethical standards of the ethics committee of the Osaka Center for Cancer and Cardiovascular Diseases Prevention and the 1964 Helsinki Declaration and its amendments. Consent for this study was obtained using the opt-out method with descriptions of the study on posters and the institutional website (www.osaka-ganjun.jp/effort/cvd/r-and-d/).

Subjects without data on drinking status (n = 57), total cholesterol (TC) (n = 1,179), or hemoglobin A1c (HbA1c) (n = 20) at baseline were excluded from the analysis. We also excluded subjects who had anemia (n = 991). Since the present study used data on height decrease per year, at least two height measurements (at baseline and end point) during observational period were necessary. Subjects without a height measurement during 2009–2019 (end point) were also excluded from the analysis (n = 3,537). The remaining 9,651 subjects with a mean age of 50.6 years (standard deviation [SD], 8.2 years; range, 40–74 years) were included in the study. The mean follow-up period of this study was 3.7 years (median, 3.0 [interquartile range, 1.9–5.6] years).

### Data collection and laboratory measurements

#### Baseline data

The baseline period of the present study was 2008–2018. Trained interviewers acquired medication history and habitual status data. Briefly, height in feet while wearing stockings and weight in while wearing light clothing were measured. BMI was calculated as weight divided by height squared (kg/m^2^). Resting blood pressure was measured twice. Mean blood pressure data was used in the analysis.

A fasting blood sample was collected. Hemoglobin (Hb), TC, triglycerides (TG), high-density lipoprotein cholesterol (HDLc), HbA1c, and serum creatinine were measured using standard procedures at the Osaka Center for Cancer and Cardiovascular Diseases Prevention. Low-density lipoprotein cholesterol (LDLc) was calculated using the Friedewald formula: LDLc = TC-(HDLc/5) mg/dL.

Between 2008 and 2012, HbA1c values were measured using the Japanese Diabetes Society (JDS) definition. Starting in 2013, HbA1c values were measured using the National Glycohemoglobin Standardization Program (NGSP) definition. The following equation, which was recently proposed by a JDS working group, was used to convert values: HbA1c(NGSP) = HbA1c(JDS) + 0.4% [20].

The World Health Organization (WHO) guidelines state that the international classification for high BMI among Asians is ≥ 25 kg/m^2^ [21]. We defined high BMI as BMI ≥ 25kg/m^2^.

Estimated glomerular filtration rate (eGFR) was calculated using an established method modified recently proposed by a working group of the Japanese Chronic Kidney Disease Initiative [22]. eGFR (mL/min/1.73 m^2^) was defined as 194 × (serum creatinine (enzyme method))^-1.094^× (age)^-0.287^ (×0.739 for women).

Chronic kidney disease was defined as eGFR < 60 mL/min/1.73 m^2^. Anemia was defined as Hb < 13 g/dL for men and Hb < 12 g/dL for women. Hypertension was defined as systolic blood pressure ≥140 mmHg, diastolic blood pressure ≥ 90 mmHg, or use of anti-hypertensive medication. Dyslipidemia was defined as TG ≥ 150 mg/dL, LDLc ≥ 140 mg/dL, HDLc < 40 mg/dL, or use of lipid-lowering medication. Diabetes was defined as HbA1c (NGSP) ≥ 6.5% or glucose-lowering medication use.

#### Endpoint data

To calculate height loss in the present study, at least 2 measurements during study period are required. Height was also measured during the endpoint period (2009–2019) using the same methods used during the baseline period (2008–2018). Briefly, height was measured while the participant was wearing stockings and light clothing.

#### Definition of height loss

Height decrease per year (mm/year) was calculated as [(height at endpoint, mm)–(height at baseline, mm)]/(follow up duration, years). Sex-specific quintile values of height decrease per year (mm/year) were also calculated. A participant was considered to have height loss if he or she was in the highest quintile of height decrease per year. For sensitivity analysis, we used another definition of height loss based on sex-specific quartiles of height decrease per year. In this sensitivity analysis, we defined height loss as being in the highest quartile of height decrease per year.

### Statistical analysis

Sex-specific characteristics of the study participants were summarized. Age, hemoglobin, and height were expressed as means ± SD. Daily drinking, current smoker status, hypertension, high BMI, dyslipidemia, diabetes, and chronic kidney disease were presented as percentages. Differences in those characteristics were calculated by sex.

Logistic regression was used to calculate odd ratios (ORs) and 95% confidence intervals (CIs) to determine associations between high BMI and height loss, high BMI and hemoglobin, and height loss and hemoglobin. For the association between height and hemoglobin, further analysis stratified by BMI status (high BMI absent versus present) was performed.

Two adjustment models were used. The first model adjusted only for age (age-adjusted model). The second model (multivariable model) also included other established confounding factors that included established cardiovascular risk factors such as drinking status (not daily drinker versus daily drinker), smoking status (no versus yes), hypertension, diabetes, dyslipidemia, and chronic kidney disease. High BMI was also included in the model used to investigate the association between height loss and hemoglobin.

To evaluate continuous values of height loss per year and hemoglobin levels by BMI status, we calculated simple correlation coefficients (r) using Spearman correlation analysis. We also calculated parameter estimates (Β) and standardized parameter estimates (β) using multiple linear regression.

All statistical analyses were performed with SAS for Windows (version 9.4; SAS Inc., Cary, NC, USA); p values of <0.05 were regarded as statistically significant.

## Results

### Characteristics of the study population by hemoglobin levels

Table 1 shows the characteristics of the study population by hemoglobin levels. For both men and women, current smoker, high BMI, and dyslipidemia were significantly positively associated with hemoglobin levels. In men, age was significantly inversely associated with hemoglobin and height was significantly positively associated with hemoglobin. In women, daily drinker status and hypertension were significantly positively associated with hemoglobin.

**Table 1:** Characteristics of the study population.

	Hemoglobin levels				*p*
	Q1	Q2	Q3	Q4	
Men					
Hemoglobin, g/dL	13.9 ± 0.4	14.8 ± 0.2	15.4 ± 0.2	16.4 ± 0.5	
No. of participants at risk	1,664	1,516	1,691	1,600	
Age, years	53.2 ± 8.3	51.3 ± 8.5	49.9 ± 8.1	49.4 ± 8.1	<0.001
Daily drinker, %	27.3	23.7	23.9	24.4	0.054
Current smoker, %	27.8	31.1	34.7	44.9	<0.001
Hypertension, %	37.3	32.3	31.3	37.0	<0.001
High BMI (BMI≥25 kg/m^2^), %	23.5	29.8	37.1	47.3	<0.001
Diabetes, %	8.3	7.2	7	10.2	0.004
Dyslipidemia, %	43.6	49.8	54.4	61.6	<0.001
Chronic kidney disease, %	10.5	9.2	9.1	10.6	0.329
Height, cm	169.6 ± 6.0	170.4 ± 5.9	170.5 ± 5.7	170.8 ± 5.9	<0.009
Women					
Hemoglobin, g/dL	12.5 ± 0.3	13.1 ± 0.1	13.6 ± 0.2	14.5 ± 0.5	
No. of participants at risk	815	756	843	766	
Age, years	50.0 ± 8.2	50.2 ± 7.9	50.3 ± 7.9	51.0 ± 7.9	0.090
Daily drinker, %	9.6	10.2	10.7	15.4	<0.001
Current smoker, %	8.7	11.0	14.5	19.2	<0.001
Hypertension, %	14.4	16.0	15.9	23.6	<0.001
High BMI (BMI≥25 kg/m^2^), %	10.1	13.4	16.6	21.0	<0.001
Diabetes, %	1.6	1.9	2.7	3.5	0.053
Dyslipidemia, %	30.1	35.3	37.8	45.3	<0.001
Chronic kidney disease, %	9.6	11.8	12.2	11.2	0.347
Height, cm	157.8 ± 5.7	157.8 ± 5.6	158.0 ± 5.4	157.6 ± 5.4	0.600

Values: Mean ± standard deviation. Quartile of hemoglobin levels for men were <14.5 g/dL for Q1 (the lowest), 14.5–15.0g/dL for Q2 (lower), 15.1–15.7g/dL for Q3 (higher), and ≥15.8 g/dL for Q4 (the highest) and for women the corresponding values were <12.9 g/dL for Q1 (the lowest), 12.9–13.3g/dL for Q2 (lower), 13.4–13.9g/dL for Q2 (higher), and ≥14.0 g/dL (the highest).

### Associations between body mass index (BMI) and height loss

S1 Table shows sex-specific associations between high BMI and height loss. For both men and women, high BMI was significantly positively associated with the incidence of height loss. This association was unchanged even after adjusting for known cardiovascular risk factors.

### Associations between high body mass index (BMI) and hemoglobin

S2 Table shows sex-specific associations between high BMI and hemoglobin. Independent of known cardiovascular risk factors, hemoglobin was significantly positively associated with high BMI for both men and women.

### Associations between height loss and hemoglobin

Table 2 shows sex-specific associations between height loss and hemoglobin. Independent from known cardiovascular risk factors, hemoglobin was significantly inversely associated with height loss for men but not for women.

**Table 2:** Odds ratios (OR) and 95% confidence intervals (CI) for height loss in relation to hemoglobin levels.

	Hemoglobin levels				p	1 SD increment of hemoglobin
	Q1	Q2	Q3	Q4		
Men						
No. at risk	1,664	1,516	1,691	1,600		
No. of cases (percentage)	388 (23.3)	306 (20.2)	315 (18.6)	285 (17.8)		
Age-adjusted ORs	Ref	0.88 (0.74, 1.04)	0.82 (0.70, 0.98)	0.79 (0.67, 0.94)	0.006	0.91 (0.86, 0.97)
Multivariable ORs	Ref	0.87 (0.73, 1.03)	0.80 (0.68, 0.95)	0.75 (0.62, 0.89)	<0.001	0.89 (0.83, 0.95)
Women						
No. at risk	815	756	843	766		
No. of cases (percentage)	161 (19.8)	158 (20.9)	168 (19.9)	149 (19.5)		
Age-adjusted ORs	Ref	1.07 (0.83, 1.37)	1.00 (0.78, 1.28)	0.93 (0.73, 1.20)	0.516	0.99 (0.90, 1.08)
Multivariable ORs	Ref	1.05 (0.82, 1.35)	0.98 (0.77, 1.26)	0.89 (0.69, 1.16)	0.341	0.97 (0.89, 1.06)

Multivariable ORs: Adjusted further for age and drinking status, smoking status, hypertension, diabetes, dyslipidemia, chronic kidney disease and high BMI. Height loss: The highest quintile of the decreased height level per year (≥ 1.79329 mm/year for men and ≥ 2.06047 mm/year for women). Quartile of hemoglobin levels for men were <14.5 g/dL for Q1 (the lowest), 14.5–15.0g/dL for Q2 (lower), 15.1–15.7g/dL for Q3 (higher), and ≥15.8 g/dL for Q4 (the highest) and for women the corresponding values were <12.9 g/dL, 12.9–13.3g/dL, 13.4–13.9g/dL, and ≥14.0 g/dL. 1 standard deviation (SD) of hemoglobin levels were 1.0 g/dL for men and 0.8 g/dL for women. Ref: Reference.

### Associations between height loss and hemoglobin by body mass index (BMI) status

Table 3 shows sex-specific associations between height loss and hemoglobin by BMI status. For men who did not have high BMI, independent from known cardiovascular risk factors, hemoglobin was significantly inversely associated with height loss. This association was not observed for men with high BMI. For women, no significant associations were observed.

**Table 3:** Odds ratios (OR) and 95% confidence intervals (CI) for height loss in relation to hemoglobin by BMI status.

	Men		Women	
	1SD increment of hemoglobin	p	1SD increment of hemoglobin	p
Not high BMI (BMI<25 kg/m^2^)				
No. at risk	4,244		2,696	
No. of cases (percentage)	799 (18.8)		512 (19.0)	
Age-adjusted ORs	0.82 (0.75, 0.89)	<0.001	0.99 (0.89, 1.09)	0.811
Multivariable ORs	0.82 (0.75, 0.89)	<0.001	0.98 (0.89, 1.09)	0.736
High BMI (BMI≥25 kg/m^2^)				
No. at risk	2,227		484	
No. of cases (percentage)	495 (22.2)		124 (25.6)	
Age-adjusted ORs	1.00 (0.91, 1.11)	0.971	0.90 (0.73, 1.10)	0.320
Multivariable ORs	1.01 (0.92, 1.12)	0.802	0.93 (0.75, 1.15)	0.520

Multivariable ORs: Adjusted further for age and drinking status, smoking status, hypertension, diabetes, dyslipidemia, and chronic kidney disease. Height loss: The highest quintile of the decreased height level per year (≥ 1.79329 mm/year for men and ≥ 2.06047 mm/year for women). 1 standard deviation (SD) increment of hemoglobin levels were 1.0 g/dL for men and 0.8 g/dL for women.

### Sensitivity analysis

To assess sensitivity, we performed the main analyses again with height loss defined as the highest quartile of height decrease per year. We obtained essentially the same results.

In the multivariable model, ORs for height loss with high BMI were 1.31 (1.15, 1.48) for men and 1.33 (1.06, 1.68) for women, respectively. In the multivariable model, ORs for high BMI and height loss for each 1 SD increase in hemoglobin were 1.46 (1.38, 1.55) and 0.91 (0.86, 0.97) for men and 1.30 (1.17, 1.44) and 1.00 (0.92, 1.08) for women. Among men stratified by BMI status, ORs for each 1 SD increase in hemoglobin and height loss were 0.84 (0.78, 0.91) for men without high BMI and 1.03 (0.94, 1.14) for men with high BMI. Among women, the corresponding values were 1.02 (0.93, 1.12) and 0.92 (0.75, 1.12), respectively.

### Correlation between continuous values of height decrease per year and hemoglobin levels by BMI status

S3 Table shows correlations between continuous values of height decrease per year and hemoglobin levels by BMI status. The simple correlation analysis showed a slight but significant inverse correlation between continuous values of height loss per year and hemoglobin only in men with BMI <25 kg/m^2^. This correlation remained even after further adjusting for known cardiovascular risk factors.

## Discussion

The major finding of the present study is that hemoglobin is significantly inversely associated with height loss in men, especially men who do not have high BMI. No significant associations were observed for men with high BMI or women.

Our previous cross-sectional study with 1,287 men aged 40–89 years revealed an inverse association between height and anemia in non-drinkers but not in drinkers [23]. Another cross-sectional study with 249 men aged 65–69 years showed a significant inverse association between height and reticulocyte count [24].

**Fig 2:**
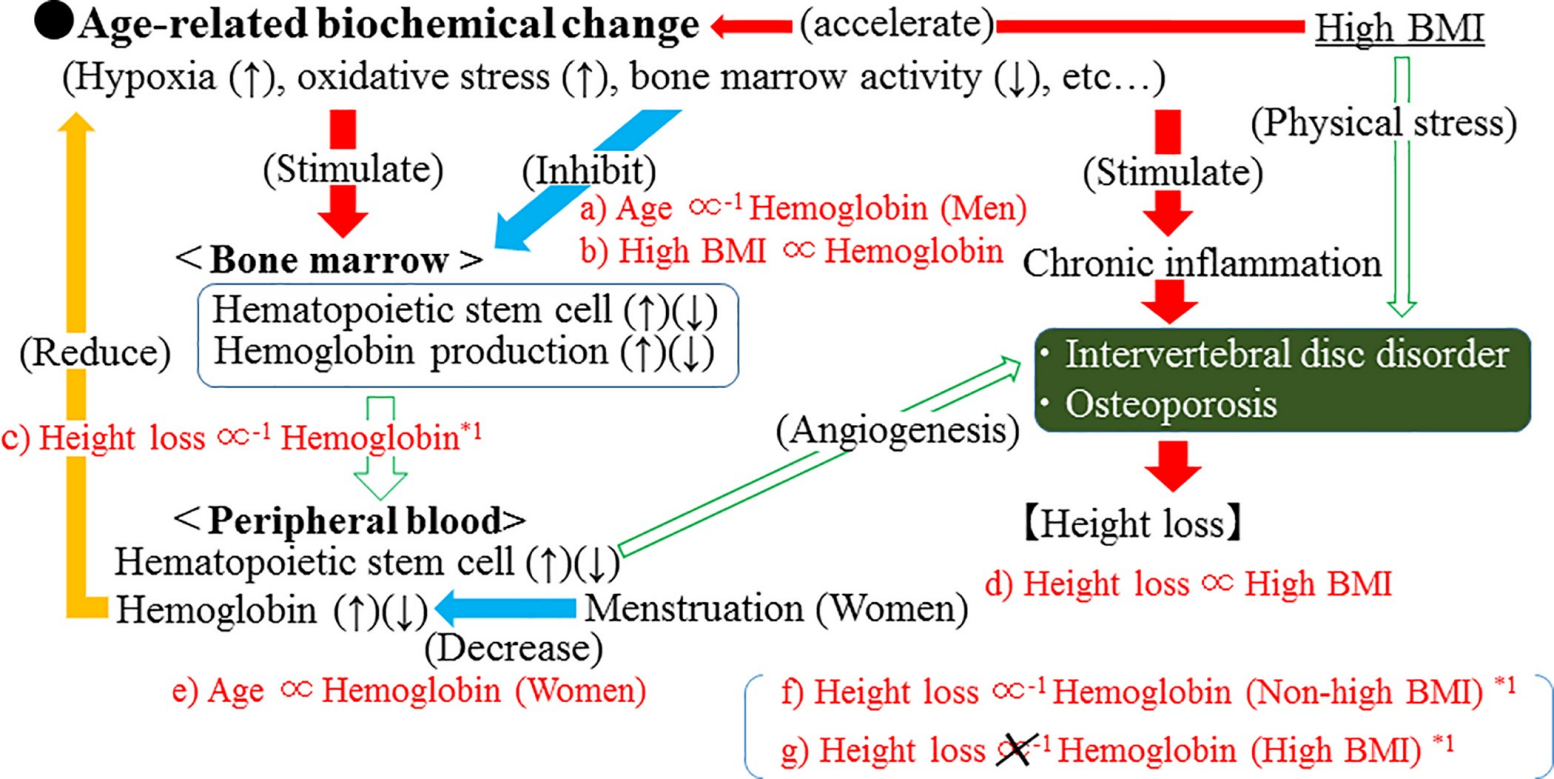
Potential mechanism underlying the association between hemoglobin and height loss. Associations shown in red (a–g) were observed in the present study. High BMI was defined as ≥25kg/m^2^. *1: Observed only among men.

In the present study, we found further evidence that hemoglobin is significantly inversely associated with height loss among Japanese men aged 40–74 years, especially those with BMI <25 kg/m^2^. However, the mechanism underlying the present results has not yet been clarified. The potential mechanisms underlying the present results are shown in Fig 2.

Aging is process that is strongly associated with increasing hypoxia and oxidative stress [2,3]. Increased hypoxia and oxidative stress have been reported to be associated with known causes of height loss such as intervertebral disc disorder [6,7] and osteoporosis [8–10]. Therefore, hemoglobin is necessary to prevent height loss. Iron deficiency, which causes anemia, is reported to accelerate intervertebral disc degeneration [25]. Older persons with osteosarcopenia were found to have low hemoglobin levels [26]. These studies partly support the argument that hemoglobin plays an important role in preventing height loss.

However, hematopoietic bone marrow activity declines with age [27,28]. Thus, hemoglobin production may be insufficient, especially in elderly individuals with increased hypoxia and oxidative stress. In this context, hemoglobin might act as an indicator of the capacity to prevent height loss. Hemoglobin found be to be inversely associated with height loss (Fig 2C).

However, this significant association was observed only in men. Differences in the influence of age on hemoglobin levels by sex might partly explain these sex-specific associations; an inverse association between hemoglobin and height loss was observed only in men. In the present study, we found a significant inverse association between age and hemoglobin; the simple correlation coefficient (r) of these two variables was -0.18 (p<0.001) (Fig 2A). For women, although menstruation might confound this association slightly, a significant positive association between these two variables was observed (r = 0.05, p = 0.008) (Fig 2E). Therefore, menstruation might act as a confounding factor on the results for women.

In the present study, for both men and women, high BMI was significantly positively associated with height loss (S1 Table, Fig 2D). The association between high BMI and fracture is inconsistent; some studies have reported positive associations while other studies have reported inverse associations [16]. A previous study reported that obesity might be associated with a lower risk of vertebral fracture [29]. However, BMI was significantly higher in subjects with disc degeneration than in subjects without disc degeneration [17]. Another study reported that obesity is associated with intervertebral disc disorder [18]. Therefore, intervertebral disk disorder might be underlying the positive association between high BMI and height loss. Both physical stress and chronic inflammation could be underlying the association between high BMI and height loss. High BMI is known to be associated with chronic inflammation [30]. A previous study reported that inflammation may play an important role in the etiology of fractures in men [31]. A strong association was observed between inflammation and intervertebral disc degeneration [32]. Further investigation with evaluation of inflammatory markers is necessary.

For both men and women, we found a significant positive association between high BMI and hemoglobin (S2 Table, Fig 2B). A previous study with 3,189 workers reported a significant positive association between hemoglobin and obesity [19], which partly supports our present results. Since chronic exposure to moderate hypoxia results in higher hemoglobin levels [33] and obesity is a state of hypoxia [34], high BMI might elevate hemoglobin levels. Obesity produces oxidative stress [35], partly via hypoxia [11]. Therefore, hemoglobin could be positively associated with high BMI, which acts as a partial indicator of oxidative stress levels.

Since high BMI was positively associated with hemoglobin (S2 Table, Fig 2B), a significant association between hemoglobin and height loss was observed in men who do not have high BMI (Table 3, Fig 2F and 2G). High BMI could act as a strong confounding factor in the association between hemoglobin and height loss.

In the present study, a significant inverse association between continuous values of height loss per year and hemoglobin levels was observed only among men without high BMI (S3 Table). However, the correlation was quite small. Diurnal height changes might act as a strong confounding factor of the influence hemoglobin has on continuous values of height loss per year [36].

One strength of the present study is that it is the first study to report that hemoglobin can prevent height loss in adult men. Based on multi-faceted analysis we could determine the potential mechanism underlying the present results because all of our present results can be explained by a simple mechanism. Declining age-related hematopoietic activity might play an important role in height loss during adulthood. Furthermore, this study showed that the concept of risk factor needs to change because among men, hemoglobin is inversely associated with height loss and positively associated with high BMI, while high BMI is significantly positively associated with height loss. Therefore, the same factor could be both beneficial and harmful and background status determines this characteristic, as in our previous studies [37,38]. Height loss starting in middle age is reported to be associated with higher total and cardiovascular mortality [1]. However, the mechanisms underlying this association are unclear. Since anemia is also reported to be associated with cardiovascular disease [39], the present study can help clarify the mechanisms underlying the association between height loss and cardiovascular disease.

The potential limitations of this study warrant consideration. In adults, height loss can be caused by vertebral fractures associated with osteoporosis and intervertebral disc degeneration, for which we did not have available data. Further investigation with data on those diseases is necessary. An efficient cutoff point to define height loss has not been established. In the present study, we used the highest quintile of height decrease per year. However, our sensitivity analysis based on quartile of height decrease per year showed essentially the same associations. Furthermore, we performed multi-faceted analysis and those results indicated a simple mechanism. Although hypoxia and oxidative stress might have a substantial effect on the study results, we had no data to evaluate oxidative stress. Further investigations with markers of hypoxia and oxidative stress such as hypoxia inducing factor (HIF), 8-hydroxydeoxyguanosine (8-OHdG), and superoxide dismutase (SOD) are necessary. Recent studies revealed a close connection between bone marrow activity and endothelial maintenance [40], including angiogenesis [41]. Since angiogenesis is also reportedly associated with intervertebral disc degeneration [42,43] and hemoglobin levels are strongly influenced by bone marrow activity [24], angiogenesis may also play an important role. However, we did not have data on angiogenesis.

## Conclusion

In men, hemoglobin is significantly inversely associated with height loss, especially in men who do not have high BMI. This association was not observed in women. These results can help clarify the mechanisms underlying height loss in adults.

## Supporting information

S1 Table Odds ratios (OR) and 95% confidence intervals (CI) for height loss in relation to BMI status. (DOCX)

S2 Table Odds ratios (OR) and 95% confidence intervals (CI) for high BMI (≥25kg/m2) in relation to hemoglobin levels. (DOCX)

S3 Table Correlation between height decrease per year (mm/year) and hemoglobin by BMI status. (DOCX)
