# Supplementary material for: Hemoglobin and adult height loss among Japanese workers: A retrospective study
Source: PLoS One. 2021 Aug 17;16(8):e0256281. doi: 10.1371/journal.pone.0256281 (PMC8370608; doi:10.1371/journal.pone.0256281)
Supplement: S1 Table — (DOCX) [file pone.0256281.s001.docx]

| **Supplemental table 1.** | | | **Odds ratios (OR) and 95% confidence intervals (CI) for height loss in relation to BMI status.** | | | |
| --- | --- | --- | --- | --- | --- | --- |
|  |  |  |  | High BMI (BMI≥25 kg/m^2^) | | p |
|  |  |  |  | (-) | (+) |  |
|  | Men | | |  |  |  |
|  |  | No. at risk | | 4,244 | 2,227 |  |
|  |  | No. of cases (percentage) | | 799 (18.8) | 495 (22.2) |  |
|  |  | Age-adjusted ORs | | Ref | 1.26 (1.10, 1.43) | <0.001 |
|  |  | Multivariable ORs | | Ref | 1.29 (1.13, 1.47) | <0.001 |
|  | Women | | |  |  |  |
|  |  | No. at risk | | 2,696 | 484 |  |
|  |  | No. of cases (percentage) | | 512 (19.0) | 124 (25.6) |  |
|  |  | Age-adjusted ORs | | Ref | 1.38 (1.10, 1.74) | 0.006 |
|  |  | Multivariable ORs | | Ref | 1.36 (1.06, 1.74) | 0.015 |
|  | Multivariable ORs: adjusted further for age and drinking status, smoking status, hypertension, diabetes, dyslipidemia, and chronic kidney disease. Height loss: The highest quintile of the decreased height level per year (≥ 1.79329 mm/year for men and ≥ 2.06047 mm/year for women). Ref: reference. | | | | | |
|  |  |  |  |  |  |  |
|  |  |  |  |  |  |  |
