# Supplementary material for: Hemoglobin and adult height loss among Japanese workers: A retrospective study
Source: PLoS One. 2021 Aug 17;16(8):e0256281. doi: 10.1371/journal.pone.0256281 (PMC8370608; doi:10.1371/journal.pone.0256281)
Supplement: S2 Table — (DOCX) [file pone.0256281.s002.docx]

| **Supplemental table 2.** | | | | | | **Odds ratios (OR) and 95% confidence intervals (CI) for high BMI (≥25kg/m2)　in relation to hemoglobin levels** | | | | | | |
| --- | --- | --- | --- | --- | --- | --- | --- | --- | --- | --- | --- | --- |
|  |  |  |  |  | Hemoglobin levels | | | | | p |  | 1 SD increment of hemoglobin |
|  |  |  |  |  | Q1 | | Q2 | Q3 | Q4 |  |  |  |
|  | Men | | | |  | |  |  |  |  |  |  |
|  |  | No. at risk | | | 1,664 | | 1,516 | 1,691 | 1,600 |  |  |  |
|  |  | No. of cases (percentage) | | | 391 (23.5) | | 452  (29.8) | 628  (37.1) | 756  (47.3) |  |  |  |
|  |  | Age-adjusted ORs | | | Ref | | 1.38  (1.18, 1.62) | 1.92  (1.65, 2.24) | 2.91  (2.50, 3.39) | <0.001 |  | 1.53  (1.45, 1.62) |
|  |  | Multivariable ORs | | | Ref | | 1.36  (1.15, 1.61) | 1.86  (1.59, 2.18) | 2.60  (2.22, 3.05) | <0.001 |  | 1.46  (1.38, 1.55) |
|  | Women | | | |  | |  |  |  |  |  |  |
|  |  | No. at risk | | | 815 | | 756 | 843 | 766 |  |  |  |
|  |  | No. of cases (percentage) | | | 82 (10.1) | | 101  (13.4) | 140  (16.6) | 161  (21.0) |  |  |  |
|  |  | Age-adjusted ORs | | | Ref | | 1.37  (1.01, 1.87) | 1.77  (1.32, 2.37) | 2.34  (1.76, 3.12) | <0.001 |  | 1.74  (1.49, 2.03) |
|  |  | Multivariable ORs | | | Ref | | 1.31  (0.95, 1.81) | 1.63  (1.20, 2.22) | 1.90  (1.40, 2.59) | <0.001 |  | 1.53  (1.30, 1.81) |
|  | Multivariable ORs: adjusted further for age and drinking status, smoking status, hypertension, diabetes, dyslipidemia, chronic kidney disease. Quartile of hemoglobin levels for men were <14.5 g/dL for Q1 (the lowest), 14.5-15.0g/dL for Q2 (lower), 15.1-15.7g/dL for Q3 (higher), and ≥15.8 g/dL for Q4 (the highest) and for women the corresponding values were <12.9 g/dL for Q1 (the lowest), 12.9-13.3g/dL for Q2 (lower), 13.4-13.9g/dL for Q2 (higher), and ≥14.0 g/dL (the highest). 1 standard deviation (SD) increment of hemoglobin levels were 1.0 g/dL for men and 0.8 g/dL for women. Ref: reference. | | | | | | | | | | | |
|  |  |  |  |  |  |  |  |  |  |  |  |  |
|  |  |  |  |  |  |  |  |  |  |  |  |  |
