# Supplementary material for: Hemoglobin and adult height loss among Japanese workers: A retrospective study
Source: PLoS One. 2021 Aug 17;16(8):e0256281. doi: 10.1371/journal.pone.0256281 (PMC8370608; doi:10.1371/journal.pone.0256281)
Supplement: S3 Table — (DOCX) [file pone.0256281.s003.docx]

| **Supplemental table 3.** | | | **Correlation between height decrease per year (mm/year) and hemoglobin by BMI status** | | | | | |
| --- | --- | --- | --- | --- | --- | --- | --- | --- |
|  |  |  |  | No. at risk | r (p) | **Multivariable** | | |
|  |  |  |  |  |  | Β | β | ｐ |
|  | Men | |  |  |  |  |  |  |
|  |  | Not high BMI (BMI<25 kg/m^2^) | | 4,244 | -0.06 (<0.001) | -0.01 | -0.04 | 0.005 |
|  |  | High BMI (BMI≥25 kg/m^2^) | | 2,227 | -0.02 (0.375) | 0.0004 | 0.001 | 0.948 |
|  | Women | | |  |  |  |  |  |
|  |  | Not high BMI (BMI<25 kg/m^2^) | | 2,696 | 0.01 (0.554) | 0.005 | 0.01 | 0.509 |
|  |  | High BMI (BMI≥25 kg/m^2^) | | 484 | -0.03 (0.501) | -0.01 | -0.03 | 0.524 |
|  | Multivariable：Adjusted for for age and drinking status, smoking status, hypertension, diabetes, dyslipidemia, and chronic kidney disease. | | | | | | | |
|  |  |  |  |  |  |  |  |  |
